# Photoluminescence Intensity Enhancement in Tin Halide Perovskites

**DOI:** 10.1002/advs.202202795

**Published:** 2022-09-15

**Authors:** Isabella Poli, Francesco Ambrosio, Antonella Treglia, Felix J. Berger, Mirko Prato, Munirah D. Albaqami, Filippo De Angelis, Annamaria Petrozza

**Affiliations:** ^1^ Center for Nano Science and Technology @PoliMi Istituto Italiano di Tecnologia via G. Pascoli 70/3 Milano 20133 Italy; ^2^ Physics Department Politecnico di Milano Piazza L. da Vinci, 32 Milano 20133 Italy; ^3^ Computational Laboratory for Hybrid/Organic Photovoltaics (CLHYO) Istituto CNR di Scienze e Tecnologie Chimiche “Giulio Natta” (CNR‐ SCITEC) Perugia Italy; ^4^ Department of Chemistry Biology and Biotechnology University of Perugia Perugia Italy; ^5^ Chemistry Department College of Science King Saud University Riyadh 11451 Saudi Arabia; ^6^ Department of Chemistry and Biology “A. Zambelli” University of Salerno Via Giovanni Paolo II 132, Fisciano Salerno 84084 Italy; ^7^ Materials Characterization Facility Istituto Italiano di Tecnologia Via Morego 30 Genova 16163 Italy

**Keywords:** Frenkel defects, lead‐free, photochemistry, photoluminescence, tin‐based perovskites

## Abstract

The prevalence of background hole doping in tin halide perovskites usually dominates their recombination dynamics. The addition of excess Sn halide source to the precursor solution is the most frequently used approach to reduce the hole doping and reveals photo‐carrier dynamics related to defects activity. This study presents an experimental and theoretical investigation on defects under light irradiation in tin halide perovskites by combining measurements of photoluminescence with first principles computational modeling. It finds that tin perovskite thin films prepared with an excess of Sn halide sources exhibit an enhancement of the photoluminescence intensity over time under continuous excitation in inert atmosphere. The authors propose a model in which light irradiation promotes the annihilation of V_Sn_^2−^/Sn_i_^2+^ Frenkel pairs, reducing the deep carrier trapping centers associated with such defect and increasing the radiative recombination. Importantly, these observations can be traced in the open‐circuit voltage dynamics of tin‐based halide perovskite solar cells, implying the relevance of controlling the Sn photochemistry to stabilize tin perovskite devices.

## Graphical abstract

Tin‐based perovskite thin films exhibit a photoinduced enhancement in the photoluminescence intensity over time due to annihilation of V_Sn_^2−^/Sn_i_^2+^ Frenkel pairs promoted by continuous illumination. Such annihilation reduces the nonradiative deep carrier trapping associated with such a defect and promotes radiative recombinations.

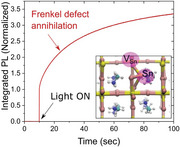

## 1 Introduction

Tin‐based perovskite semiconductors can reach a bandgap of 1.3–1.4 eV and, in theory, power conversion efficiencies (PCE) higher than 30%.^[^ ^1^ ^]^ In practice, devices with efficiencies below 10% are mostly reported in the literature,^[^ ^2^, ^3^, ^4^, ^5^, ^6^ ^]^ with a champion PCE of 14.7%.^[^ ^7^ ^]^ The easy oxidation of Sn(II), due to the inherently low potential of the Sn(II)/Sn(IV) couple of only 0.15 V versus SHE (standard hydrogen electrode),^[^ ^8^ ^]^ induces the formation of Sn vacancies, which act as acceptor levels,^[^ ^9^ ^]^ positively doping the material and severely limiting the diffusion carrier length.^[^ ^10^ ^]^ Moreover, p‐doping introduced by tin vacancies can promote the oxidation of Sn(II) to Sn(IV) at the surface. Sn(IV) defects act as electron traps and favor lattice degradation.^[^ ^11^ ^]^ Indeed, pristine tin halide perovskite solar cells show low efficiencies and reproducibility and Sn(IV) content is believed to be one of the main factors limiting the photovoltaic performance of Sn‐based perovskite solar cells.^[^ ^12^ ^]^ Therefore, it is crucial to control the p‐doping of Sn perovskites introduced by tin vacancies to limit the formation of Sn(IV) defects which increase nonradiative recombination.

Different strategies have been explored to reduce the self p‐doping of tin containing perovskites, such as the use of reducing agents, like hypophosphorous acid and Sn(0) nanoparticles,^[^ ^13^, ^14^, ^15^ ^]^ the identification of alternative solvents that do not induce Sn(II) oxidation^[^ ^16^ ^]^ and the incorporation of bulky ammonium cations.^[^ ^17^, ^18^ ^]^ One of the foremost used approaches found in the literature looks at the addition of extra tin halide (SnF_2_, SnCl_2_, or excess SnI_2_) within the precursor solution to reduce the probability of formation of Sn vacancies during film crystallization, therefore reducing the background doping.^[^ ^9^, ^19^, ^20^, ^21^, ^22^, ^23^ ^]^ Despite the progresses achieved in tin halide perovskites, there are still many open questions about the nature and role of defects on the optoelectronic properties and stability of the material. Two types of carrier traps have been predicted in these systems: shallow traps, related to tin vacancy and halide interstitial defects,^[^ ^9^ ^]^ which contribute to the p‐doping of the material, and deep traps, introduced by Sn interstitials and halide vacancies, that may play a role in nonradiative recombination processes. These two classes of defects and the relative optoelectronic dynamics should be decoupled, however, the high background doping concentration in tin halide perovskites may increase the radiative efficiency of the material, masking other optoelectronic nonradiative recombination processes.^[^ ^24^, ^25^ ^]^

The addition of excess tin halide in tin perovskites limits the density of background holes, which reduces the radiative rate and opens up the chance for nonradiative recombination channels to become effective, especially at low to medium injection levels. In this work, we report an experimental and theoretical investigation of the defect activity in tin halide perovskite thin films prepared with excess Sn halide by combining the photoluminescence evolution over time under continuous optical excitation with first principles computational modeling of defect formation under light irradiation. The photoluminescence is extremely sensitive to recombination rate constants, defect densities, and nonradiative decay paths; therefore, we can take it as a measure of the optoelectronic quality of the material. We show that, as long as the doping density is relatively low, i.e., in Sn perovskite films with addition of excess tin halide, a photoluminescence intensity enhancement phenomenon is observed under continuous photoexcitation. We propose a model in which light irradiation promotes the annihilation of V_Sn_^2−^/Sn_i_^2+^ Frenkel pairs, which we show to be relatively abundant in Sn halide perovskite materials, thus partly restoring a more perfect crystalline environment and reducing the trapping centers associated with such defects.

## 2 Results and Discussion

**Figure 1:**
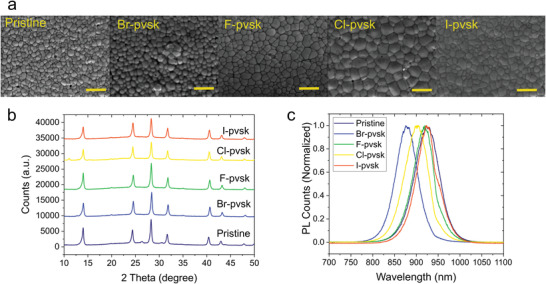
a) Top‐view SEM images of FACsSnI films without and with addition of excess SnX_2_. Scale bar = 2 µm. b) XRD patterns of FACsSnI thin films. c) Photoluminescence spectra of FACsSnI thin films measured under 450 nm excitation at 100 mW cm^−2^ intensity. All films have been encapsulated and measured in air.

We prepare pristine FA_0.85_Cs_0.15_SnI_3_ (FACsSnI) thin films (Pristine) and FACsSnI with 10 mol% addition of excess SnX_2_ with X = Br, Cl, F, and I (Br‐pvsk, Cl‐pvsk, F‐pvsk, and I‐pvsk). The addition of excess SnX_2_ creates a Sn‐rich condition of growth that aims at hindering the formation of tin vacancies increasing the chemical potential of tin, while the pristine film grows under Sn‐medium conditions.^[^ ^9^, ^26^ ^]^ **Figure** **1**a shows the top‐view scanning electron microscope (SEM) images of FACsSnI films without and with additives (see Figure S1, Supporting Information, for large area SEM images). All films show good coverage and homogeneous grain size. FACsSnI films that contain excess SnX_2_ exhibit larger grains, particularly the Cl‐pvsk sample, which shows an average grain size higher than 1 µm (see Figure S2, Supporting Information). According to the X‐ray diffraction (XRD) patterns shown in Figure 1b, all films exhibit a pseudocubic crystal structure (Pm3m), with typical perovskite peaks at 2*θ* ≈ 14°, 24.5°, 28.3°, 31.6°, 40.4°, 42.8°, and 47.8°, which correspond to the (100), (111), (200), (210), (220), (300), and (311) directions, respectively.^[^ ^27^ ^]^ The Cl‐pvsk film shows a reflection at 2*θ* = 11°, which can be assigned to unconverted SnCl_2_.^[^ ^28^ ^]^ Furthermore, compared to the pristine perovskite, the X‐ray diffraction peaks of the Br‐ and Cl‐pvsk thin films slightly shift to higher angles, suggesting that I^−^ is partially substituted with Cl^−^ and Br^−^ causing a slight contraction of the unit‐cell volume (Figure S3, Supporting Information). The photoluminescence spectra of FACsSnI thin films are shown in Figure 1c, showing PL peak maxima ranging between 880 and 930 nm, which correspond to bandgaps of 1.33–1.4 eV. Br‐pvsk and Cl‐psvk spectra are blueshifted with respect to the others, indicating the partial incorporation of the smaller halogen into the crystal lattice, as previously observed in the literature.^[^ ^29^, ^30^ ^]^ F‐pvsk does not show any PL peak shift with respect to the pristine film, however we confirm the presence of F on the surface by measuring the X‐ray photoelectron spectroscopy (XPS) F1s spectra of F‐pvsk and pristine FACsSnI thin films (Figure S4, Supporting Information).

**Figure 2:**
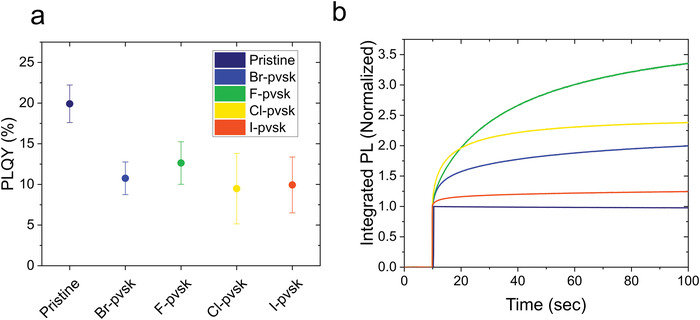
a) Absolute PLQY values of pristine FACsSnI and films with addition of excess SnX_2_ (each signal is measured with an integration time of 10 s and 10 scans are averaged for each signal). The average PLQY values of pristine, Br‐pvsk, F‐pvsk, Cl‐pvsk, and I‐pvsk FACsSnI films are measured over a set of 7, 3, 9, 3, and 3 samples, respectively; all samples come from different fabrication batches. The error bars indicate the 95% confidence interval. b) Integrated photoluminescence intensity overtime, normalized with respect to the value measured at time 0 under illumination. Excitation light: 450 nm at 100 mW cm^−2^.

In order to get an insight into the optoelectronic properties of the materials and investigate the effect that excess SnX_2_ has on the defect chemistry, we monitored the photoluminescence intensity of FACsSnI thin films without and with additives. To exclude the effect of oxygen, all films were encapsulated in the glove box right after deposition under sub‐ppm oxygen concentrations. The photoluminescence quantum yield is the ratio between emitted photons per molecular excitation and is an essential parameter for the primary optoelectronic characterization of electroluminescent devices like perovskite solar cells. **Figure** **2**a shows the absolute PLQY values of FACsSnI thin films without and with addition of excess SnX_2_ measured at 100 mW cm^−2^ with 375 nm excitation. FACsSnI samples that contain SnX_2_ additives have similar PLQY of about 10%–12%, while the pristine‐FACsSnI film shows an absolute value of about 20%, indicating that a higher fraction of photogenerated carriers recombines radiatively when compared to FACsSnI with excess SnX_2_. Such high radiative efficiency in pristine tin halide perovskites is due to the intense background hole doping, which introduces pseudo‐monomolecular radiative recombination pathways between the photoexcited electrons and background holes.^[^ ^24^, ^31^ ^]^ The addition of excess SnX_2_ creates a tin‐rich environment, which reduces the likelihood of tin vacancy formation and hole doping. Indeed, we observe that adding excess SnX_2_ to FACsSnI thin films reduces the conductivity by over an order of magnitude (Figure S5, Supporting Information). We also quantify the doping levels of pristine and F‐pvsk FACsSnI thin films via Hall effect measurements, measuring a doping concentration of 6 × 10^18^ and 2 × 10^17^ cm^−3^, respectively, confirming that the addition of SnF_2_ reduces the background hole doping density. Theoretical studies on tin halide perovskites suggested that tin‐rich environment may also contribute to the formation of nonradiative recombination‐active deep traps, such as tin interstitials and iodide vacancies.^[^ ^9^ ^]^ As we recently showed experimentally, ^[^ ^25^ ^]^ the reduced PLQY observed in FACsSnI thin films with excess SnX_2_ with respect to the pristine sample can be explained by a lower background hole doping, which reduces the pseudo‐monomolecular radiative recombination, and a higher probability of formation of deep‐level traps, which contribute to nonradiative recombination processes.

Figure 2b shows the integrated photoluminescence over time of thin films deposited on glass and excited with a 450 nm laser beam incident on the film surface. The values have been normalized by the initial intensity measured as soon as the laser beam is turned on (the absolute values are shown in Figure S6, Supporting Information). We observe that the pristine FACsSnI thin film is highly stable under continuous illumination, while FACsSnI films with excess SnX_2_ show a consistent photoluminescence intensity enhancement without detectable shifts in the PL peak position, suggesting the absence of photoinduced phase segregation phenomena (Figure S7, Supporting Information). The phenomenon remains active also when the substrates are heated at 55 and 85 °C (Figure S8, Supporting Information) and the same photoluminescence enhancement is observed when the absolute PLQY is monitored under illumination within the integrating sphere (Figure S9, Supporting Information). Most importantly, no variation in the conductivity of the films was observed under illumination, suggesting that the background hole doping density is not considerably changing under light (Figure S10, Supporting Information). Therefore, we expect a variation in deep traps, which do not contribute to doping, to be responsible for the observed PL intensity change under illumination. The reason why pristine FACsSnI thin films do not exhibit the same PL intensity healing phenomenon observed in FACsSnI with excess SnX_2_ might be due to a higher background doping that makes pseudo monomolecular radiative recombination processes due to doping dominating over nonradiative recombination.

**Figure 3:**
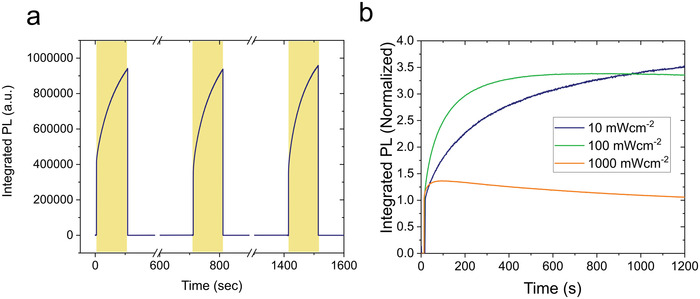
a) Integrated photoluminescence of a F‐pvsk thin film measured with an excitation of 450 nm. The film was initially illuminated for about 2 min. The photoluminescence was measured again in the same spot after 10 min of resting in the dark. The cycle was repeated two times. b) Integrated photoluminescence of a F‐pvsk thin film excited with a 450 nm under 10, 100, and 1000 mW cm^−2^ power densities.

To identify which additional factors affect the photoresponse of FACsSnI thin films, we investigate the photoluminescence evolution under light/dark cycles and different excitation densities (**Figure** **3**). The integrated photoluminescence intensity under light‐dark cycles (2 min under light followed by 10 min in the dark) of a F‐pvsk FACsSnI thin film suggests that the photoluminescence healing phenomenon observed under light is fully reversible and that initial conditions are recovered when the sample is kept in the dark (Figure 3a). Similar trends are observed for Br‐pvsk, Cl‐pvsk, and I‐pvsk samples (Figures S11–S13, Supporting Information), while pristine FACsSnI films show stable PL under light‐dark cycles (Figure S14, Supporting Information). Figure 3b shows the integrated photoluminescence intensity of a F‐pvsk thin film measured with an excitation of 450 nm at 10, 100, and 1000 mW cm^−2^. When measuring the sample under low excitation densities (10 mW cm^−2^) the photohealing effect slows down without reaching a plateau in 20 min of continuous illumination. In contrast, when the photoluminescence is monitored under high excitation densities (1000 mW cm^−2^), an initial fast healing is followed by a quenching effect over time. Similar PL enhancement and quenching effects under different excitation light are observed on Br‐pvsk and Cl‐pvsk samples too, as shown in Figures S15 and S16 (Supporting Information), respectively.

**Figure 4:**
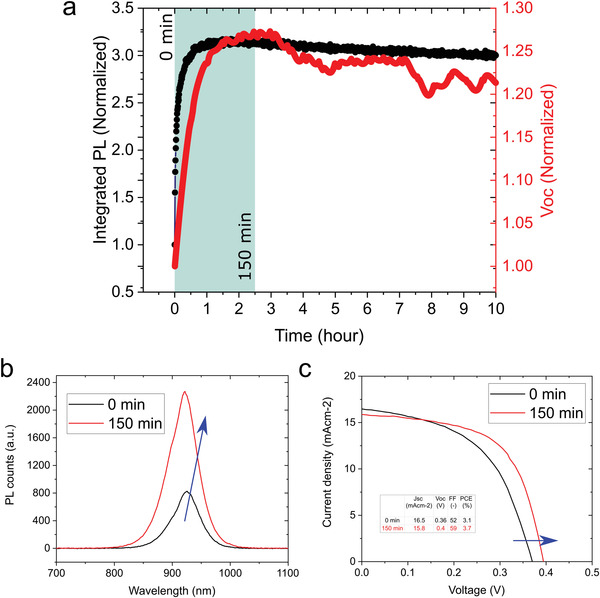
a) Evolution of the integrated photoluminescence of a F‐pvsk FACsSnI thin film and the open‐circuit voltage of a F‐pvsk FACsSnI based solar cell under continuous 1 sun illumination (cell kept at open circuit). The PL is measured by continuously exciting the encapsulated film with a single‐wavelength 525 nm laser (2.5 mW) at 25 °C. The values have been normalized by the initial value measured as soon as the laser beam/solar simulator is turned on. b) PL peak of a F‐pvsk FACsSnI thin film before and after 150 min of continuous excitation (525 nm laser, 2.5 mW, 25 °C). c) *JV* curve of a F‐pvsk FACsSnI solar cell before and after 150 min of continuous illumination (simulated 1 sun illumination, *V*_OC_ condition, 25 °C).

In perovskite solar cells, like other photovoltaic devices, maximizing the external quantum efficiency is a target to achieve. The photovoltaic parameter that is mainly limited by nonradiative recombination occurring both within the perovskite layer and at the perovskite/extraction layers’ interfaces is the open circuit voltage (*V*_OC_).^[^ ^32^ ^]^ To explore the effect that the observed photoinduced PLQY healing might have on the performance and stability of full photovoltaic devices, we also fabricate F‐pvsk solar cells and keep track of the open‐circuit voltage under continuous simulated 1 sun illumination for 10 h. To directly relate the PL and *V*_OC_ evolution under illumination, we also monitor the photoluminescence spectrum of an encapsulated F‐pvsk thin film under continuous single‐wavelength excitation for the same amount of time (525 nm, 2.5 mW). **Figure** **4**a shows the integrated PL intensity and the *V*_OC_ of the F‐pvsk thin film and device, respectively. The photoluminescence peak of the thin film becomes almost three times more intense (Figure 4b) and the efficiency of the device increases from 3.1% to 3.7% (Figure 4c) during the first 150 min of illumination. The PL intensity and open‐circuit voltage exhibit a very similar dynamic under illumination, where an initial considerable increase is followed by a much slower decrease, suggesting that the enhancement of the open‐circuit voltage is correlated to a reduction of nonradiative recombination within the perovskite layer.

The timescales involved in the photoluminescence enhancement and quenching observed herein on tin halide perovskites are extremely slow, tens of seconds, and consistent with the timescale of ionic activities, such as ion/defect annihilation and migration rates and that of photoinduced transformations in the archetypal MAPbI_3_ (MA = methylammonium).^[^ ^33^, ^34^, ^35^, ^36^ ^]^ Therefore, we believe that, similarly to what was previously observed on MAPbI_3_,^[^ ^35^ ^]^ a sequence of ion reorganization and migration effects could be activated in tin halide perovskites too, leading to the annihilation of Frenkel pairs under illumination and subsequent photoluminescence enhancement effect. In order to investigate this hypothesis, we perform advanced electronic‐structure calculations on the archetypal tin perovskite MASnI_3_ (cf. Computational Details). In this regard, we note that previous studies have shown that the effect of the A‐site cation on the defect physics and charge localization in metal halide perovskites is exiguous when considering cations with similar size.^[^ ^37^, ^38^ ^]^

**Figure 5:**
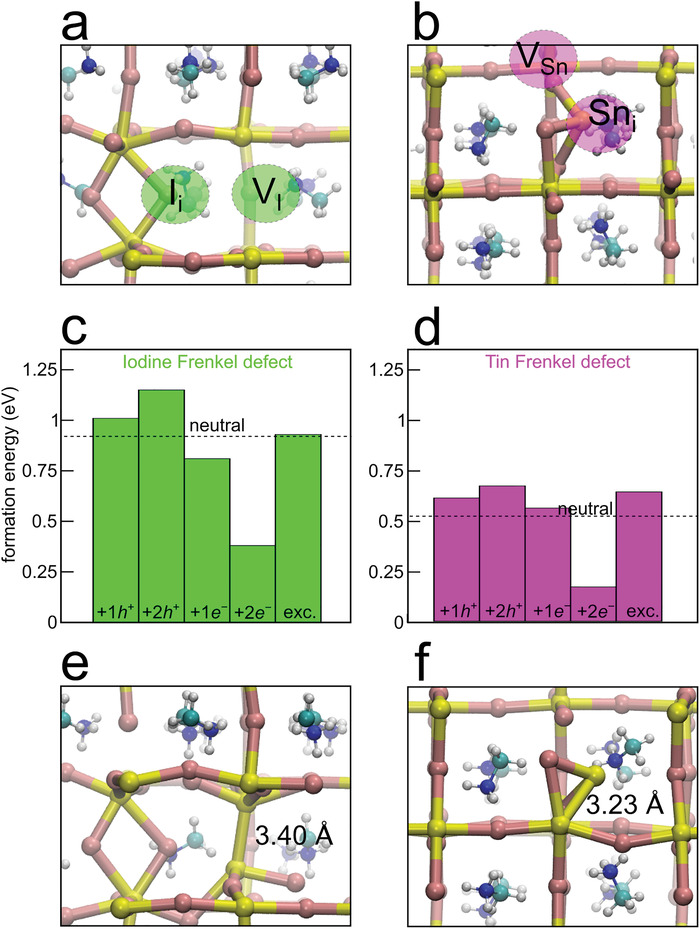
a,b) Stick & ball representation of neutral iodine and tin Frenkel defects, respectively. Sn in yellow, I in pink, C in cyan, N in blue, and H in white. c,d) Formation energies of iodine and tin Frenkel defects with respect to the bulk MASnI_3_, respectively. The formation energies of the neutral defects are reported as dashed lines. e,f) Structural configurations featuring electron localization via the formation of a Sn—Sn bond for iodine and tin Frenkel defects, respectively.

For tin iodide perovskites, it has been shown that, under Sn‐rich conditions, as those achieved here by adding an excess of SnX_2_, self p‐doping can be limited by the formation of Frenkel defects.^[^ ^9^ ^]^ In order to verify a possible role of these defects in the observed photohealing process, we first model the neutral Frenkel defects (FD) associated with iodine and tin vacancy/interstitial, i.e., $V_{I}^{+}/I_{i}^{-}$ and $V_{\mathrm{Sn}}^{2-}/{\mathrm{Sn}}_{i}^{2+}$ pairs (**Figure** **5**a,b for details of the structural models). From the total‐energy difference between the defective models and the pristine bulk, we calculate defect formation energies of 0.92 and 0.52 eV for $V_{I}^{+}/I_{i}^{-}$ and $V_{\mathrm{Sn}}^{2-}/{\mathrm{Sn}}_{i}^{2+}$, respectively (cf. dashed lines in Figure 5c,d), to be compared with the values of 1.09 and 1.01 eV calculated for the noninteracting defects as calculated in two different supercells.

In order to assess how photo‐generated charges might change the energetics of Frenkel pairs, we consider: i) injection of one and two extra holes on hole‐accepting I_i_ and V_Sn_, ii) injection of one and two extra electrons on electron accepting V_I_ and Sn_i_, and iii) excited state simulated by imposing a triplet spin multiplicity to the supercell. For each considered system, we recalculate the defect formation energy with respect to the pertinent charged or excited bulk model. Results are collected in Figure 5c,d. For both Sn and I Frenkel defects (SnFD and IFD), the injection of extra holes results in a mild destabilization of the pair with increased formation energies up to 0.1–0.2 eV. This is consistent with the poor hole‐trapping properties of both I_i_ and V_Sn_ in the bulk tin perovskite with charge transition levels below the valence band edge of the material^[^ ^9^ ^]^ and here calculated also for the Frenkel defects (cf. Figure S17, Supporting Information). Upon injection of a single extra electron, we observe a small effect for both the Frenkel defects, with either slight destabilization (+0.04 eV with respect to the neutral case) for the SnFD or small stabilization (− 0.09 eV) for the IFD. We notice that, for both V_I_ and Sn_i_ in tin perovskites, it is known that an extra electron can localize upon formation of a Sn—Sn bond.^[^ ^9^, ^37^ ^]^ In particular, we find the structural configurations featuring a Sn‐Sn bond of 3.40 and 3.23 Å for the iodine and the tin defect, respectively, (Figure 5e,f) to be almost isoenergetic (<0.05 eV difference) with those entailing charge delocalization. However, when a second electron is added to the defective system, charge trapping on the Sn—Sn moiety is strongly advantaged with respect to electron delocalization, as we observe Sn—Sn bonds shrunk to 3.22 and 2.90 Å and we calculate an energy gain of 0.38 and 0.43 eV, with respect to the delocalized electrons, for IFD and SnFD, respectively. The formation energies of FD are then drastically lowered with respect to the neutral case (Figure 5a,b) and the associated charge transition levels lie deep in the band gap of the material (Figure S17, Supporting Information).

Finally, we investigate the energetics of FDs in the photoexcited state, which is most representative of the conditions in which PL experiments are conducted. For the IFD, we find no significant difference in the formation energy (<0.01 eV) with respect to the neutral ground state, a result essentially stemming from the opposite contributions calculated for the isolated unpaired hole and electrons. This result, in conjunction with the lower concentration of IFDs predicted from formation energies, suggests that the observed photo‐healing effect is not likely to originate from photo‐induced annihilation of the IFD. In contrast, the SnFD is found to be significantly destabilized by 0.15 eV when considering the excitonic state. Therefore, annihilation of this defect under illumination may contribute to the time‐dependent evolution of the PL for samples in which the self p‐doping of the tin perovskite induced by Sn vacancies is alleviated, i.e., when the material is synthetized with the addition of SnX_2_.

**Figure 6:**
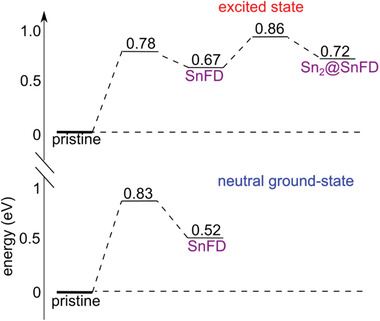
Energy diagrams for the tin Frenkel defect in the neutral ground state (bottom panel) and in the excited state (top panel). In each diagram, energies are referred to that of the pristine bulk material, which is highlighted with a thicker horizontal line.

To investigate the possible mechanism of photoinduced annihilation of the Sn Frenkel pair, we carry out linear transit calculations between the pristine and the defective supercell for structural configurations achieved at both the ground and the excited state. We observe that annihilation of the Frenkel pair in the ground state is hindered by a substantial energy barrier of 0.31 eV (**Figure** **6**). However, the energy profile of the excited state reveals that not only the SnFD is destabilized but also the energy barrier associated with annihilation is drastically reduced to 0.11 eV (Figure 6). Therefore, the measured PL change can be explained with the photo‐annihilation of the Sn Frenkel pair, which is induced by the synergistic effect of destabilization of the defect in the excited state and a reduced energy barrier for the process, with respect to the neutral ground state. When the illumination is interrupted, the system returns to the equilibrium ground‐state and Sn Frenkel pairs can reappear over time as the formation energy is lower, thus explaining the reversibility of the process.

We pinpoint that the most stable structural model of SnFD in the excited state does not feature electron‐trapping. However, a structural model bearing a Sn—Sn dimer is found at an energy only 0.05 eV higher (Figure 6). From linear transit calculations, the formation of the Sn—Sn moiety may occur by overcoming an energy barrier of 0.19 eV, which is almost double of that calculated for defect annihilation (Figure 6), thus indicating that a pathway leading to a self‐trapped exciton is kinetically hindered. However, localization of a second electron, which can be possible at high density of photogenerated carriers, may strongly stabilize the dimer (Figure 5d), which would then act as deep recombination center. Furthermore, in this case detrapping would be amply disadvantaged by an energy barrier of 0.59 eV for the release of the localized electrons. Such a result is in line with the observed drop in PL when the sample is irradiated with light at high intensity.

## 3 Conclusions

We have studied the evolution of the emissive properties of tin halide perovskites under continuous excitation by combining photoluminescence measurements and computational calculations. We report a light induced photoluminescence intensity enhancement in tin halide perovskite thin films, which is consistent with a reduction of nonradiative decay pathways. We demonstrate that the phenomenon is light intensity dependent and is observed in materials synthetized with addition of SnX_2_, irrespectively of the halogen used (F, Br, Cl, or I), where the self p‐doping induced by Sn vacancies is alleviated. We propose a model in which light irradiation promotes the annihilation of Sn vacancy/interstitial Frenkel pairs, thus partly restoring a better crystalline environment and reducing some of the trapping centers associated to such defects. Annihilation of Frenkel pairs is induced by the synergistic effect of destabilization of the defect in the excited state and a reduced energy barrier for the process. We show that high‐intensity light irradiation, modeled as the localization of a second electron, stabilizes Sn vacancy/interstitial Frenkel defects, which would then act as deep recombination centers, supporting the PL intensity quenching experimentally observed when Sn films are illuminated with 1000 mW cm^−2^ power densities. Finally, we show that the *V*_OC_ of tin halide perovskite solar cells with SnF_2_ excess mirrors the PL intensity healing effect under 1 sun illumination, unveiling the relevance of controlling the tin photochemistry toward long‐term stabilization of Sn perovskite devices.

## 4 Experimental Section

### Materials and Fabrication

N,N‐dimethylformamide (DMF, anhydrous, 99.8%), dimethyl sulfoxide (DMSO, anhydrous, ≥99.9%), and anisole (anhydrous, 99.7%) were purchased from Sigma‐Aldrich; tin(II) iodide (SnI_2_, for perovskite precursor) was purchased from Tokyo Chemical Industry (TCI); tin(II) fluoride (SnF_2_, 97.5%) was purchased by Alfa Aesar. All chemicals were used without any further purification. Glass substrates were cleaned in acetone and isopropyl alcohol (IPA) for 10 min by sonication. The cleaned glass substrates were treated with oxygen plasma for 10 min before any further deposition. Thin‐film perovskite deposition was done in a N_2_‐filled glovebox and thin films were glass encapsulated immediately after thermal annealing (in the glovebox to avoid oxygen).

### Sn‐Based Thin Films

To make Sn‐based (FA_0.85_Cs_0.15_SnI_3_) thin‐film perovskite the precursor solution (concentration of 1.2 m) was prepared in mixed solvents of DMF and DMSO with a volume ratio of 4:1. The molar ratio for FAI/CsI was 0.85:0.15 and the molar ratio of (FAI+CsI)/ SnI_2_ was 1:1. 10 mol% of SnX_2_ (X = F, Br, Cl, I) was added in the precursor solution. The precursor solution was stirred at 40 °C for 30 min and then filtered through 0.20‐µm PTFE membrane before use. The perovskite films were deposited with one‐step spin‐coating procedures at 4000 rpm for 50 s. Anisole (80 µL) was dropped on the spinning substrate 25 s before the end of the procedure. The substrates were annealed at 120 °C for 20 min.

### Sn‐Based Solar Cells

Patterned ITO glass was cleaned in detergent, deionized water, acetone, and isopropanol sequentially by ultrasonics and then treated with UV–ozone for 10 min. A thin layer of poly(3,4‐ethylene‐dioxythiophene):polystyrene sulfonate (PEDOT:PSS) was deposited by spin‐coating the PEDOT:PSS solution (Clevios P VP AI 4083), after filtration through a PVDF 0.45 µm pore size filter, onto ITO substrates at 5000 rpm for 30 s, followed by 10 min annealing in air at 150 °C. A perovskite Sn‐based thin film was deposited on top of PEDOT:PSS by using a 0.3 m concentrated Sn solution and spin‐coated at 5000 rpm for 50 s. Anisole was dropped onto the spinning substrate after 25 s from the start. A thin layer of [6,6]‐phenyl‐C61‐butyricacid methyl (PCBM) was deposited onto the perovskite layer by spin coating a PCBM solution (20 mg mL^−1^ in chlorobenzene) at 2000 rpm for 30 s. A solution of bathcuproine (BCP) of 0.5 mg mL^−1^ in anhydrous propan‐2‐ol was spin‐coated dynamically onto PCBM at 4000 rpm for 30 s. Finally, 80 nm of Ag was thermally evaporated.

### Characterization

The current density–voltage (*J–V*) characteristics were recorded with a Keithley 2440. The illuminated electrode area, defined with a holed black anodized aluminium mask, was 0.0935 cm^2^. Encapsulated devices were measured in an ambient atmosphere at 23 ± 2 °C and 40%–60% relative humidity. The scan rates of the *J*–*V* sweep were 0.13 V s^−1^.

Stability tests were performed by means of a P&O Tracker (Arkeo, Cicci Research), under simulated 1 sun illumination (provided by LED lights), in N_2_ atmosphere, at open‐circuit conditions, and at 25 °C. UV filters were not applied.

XRD patterns were recorded with a Bruker D8 Advance diffractometer with Bragg–Brentano geometry equipped with a Cu K*α*1 (*λ* = 1.544060 Å) anode, operating at 40 kV and 40 mA. All the diffraction patterns were collected at room temperature, with a step size of 0.05 in symmetric scan reflection mode, an acquisition time of 1 s and within a Bruker airtight specimen holder with dome like X‐ray transparent cap,.

SEM images were obtained using a MIRA3 TESCAN microscope with an accelerating voltage of 5 kV. Perovskite films were prepared on ITO substrates.

Steady‐state PL: The excitation source was an unfocused beam of a 450 nm c.w. diode laser (Oxxius) or a 525 nm c.w. diode laser (Roithner Lasertechnik GmbH). Photoluminescence was collected in reflection mode at a right angle from the excitation line and focused into a fiber coupled to a spectrometer (Ocean Optics Maya Pro 2000) with an intensity of ≈100 mW cm^−2^. PL was measured in air on glass encapsulated samples.

Absolute PLQY measurements were obtained from measurements performed in an integrating sphere (Labsphere) on encapsulated thin films deposited on nonconductive glass. Excitation was provided by a 375 nm c.w. diode laser (beam diameter ≈ 370 nm) and spectra acquired through an optical fiber coupled from the sphere to a spectrometer (Ocean Optics Maya Pro 2000) with an excitation power of 100 mW cm^−2^. PLQY values were calculated using the method proposed by de Mello et al.^[^ ^39^ ^]^ Error bars indicate 95% confidence intervals.

Electrical conductivity (*σ*) measurements were obtained by depositing the perovskite film onto Au gold stripe contacts. *σ* was calculated as $\sigma =\frac{/}{Rwt}$, where *l* is the length of the Au contacts, *R* is the average resistance, *t* is the thickness of the perovskite film, and *w* is the width between the two Au contacts. The resistance *R* was measured by using a two‐point electrical probe. An Agilent B1500A Semiconductor Device Parameter Analyzer (SPA) was used to impose a voltage sweep from −1 to 1 V between the two probes and the corresponding values of current were recorded.

Hall effect measurements were obtained using a Hall effect measurement system (semiautomatic) (HMS5300, Ecopia) using Van Der Pauw method with constant current source and 0.51 Tesla permanent magnet.

XPS was carried out on a Kratos Axis Ultra^DLD^ spectrometer. High‐resolution spectra were acquired at a pass energy of 10 eV using a monochromatic Al K*α* source (15 kV, 20 mA). XPS data were analyzed using CasaXPS (version 2.3.24).^[^ ^40^ ^]^

### Computational Details

To simulate Frenkel defects in bulk MASnI_3_, a 2 × 2 × 2 supercell (*a* = *b* = 17.5154 Å, *c* = 24.8580 Å) corresponding to the experimental density was used. Hybrid DFT calculations were carried out at the PBE0+rVV10 level of theory,^[^ ^41^, ^42^, ^43^, ^44^ ^]^ in line with previous publications.^[^ ^45^, ^46^, ^47^ ^]^ Nonlocal van der Waals interactions were included through the rVV10 scheme, in which the *b* parameter governing the extent of long‐range interactions is set to its original value of 6.3.^[^ ^41^, ^42^ ^]^ All calculations were carried out with the freely available CP2K suite of codes. Goedecker–Teter–Hutter pseudopotentials were used to account for core‐valence interactions.^[^ ^48^ ^]^ Double‐*ζ* polarized basis sets were used for the wave functions and a cut‐off of 600 Ry for the expansion of the electron density in plane waves.^[^ ^49^ ^]^ The auxiliary density matrix method was used to speed up the calculation of exact exchange in hybrid functional^[^ ^50^ ^]^ calculations as implemented in CP2K with the cFIT auxiliary basis set.^[^ ^51^ ^]^ Spin‐polarized calculations were performed on systems bearing an odd number of electrons. Calculations of energy barriers for hole trapping on interstitial iodide were carried out using a modified version of the linear transit method^[^ ^52^ ^]^ previously used by Ambrosio et al.^[^ ^53^ ^]^ The coordinates of the two structures *R*_*i*_ and *R*_*j*_ were linearly interpolated^[^ ^52^ ^]^ according to the following expression: *R*_*λ*_ = *λR*_*i*_ + (1 − *λ*)*R_j_* where *λ* is the coupling parameter connecting the two models. The achieved structures were then allowed to undergo structural relaxation in which the organic cations are free to relax. In contrast, the positions of the atoms belonging to the inorganic sublattice were fixed. In this way, unstable and highly energetic structures can be avoided due to linear interpolation of the coordinates of the freely rotating organic cations. The accuracy of this methodology was checked in previous studies.^[^ ^47^, ^53^, ^54^ ^]^ Charge transition levels of the Frenkel defects reported in Figure S16 (Supporting Information) were calculated using the grand‐canonical formulation of defects in crystalline materials^[^ ^55^ ^]^ and are defined as

(1) $$\mu \left(q/q^{\prime }\right)=\frac{E^{q}\left[X\right]-E^{q^{\prime }}\left[X\right]}{q^{\prime }-q}+\frac{E_{corr}^{q}-E_{corr}^{q^{\prime }}}{q^{\prime }-q}-\varepsilon_{V}$$

where $E^{q}\left[X\right]$ and $E^{q^{\prime }}\left[X\right]$ are the total energies of the defect *X* in the charge state *q* and *q*′, respectively, *ε*_V_ the valence band edge of the pristine perovskite, and $E_{\mathrm{corr}}^{q}$ and $E_{corr}^{q^{\prime }}$ correction term introduced to account for electrostatic finite‐size effects of charged periodic supercells and here calculated with the scheme proposed by Freysoldt, Neugebauer, and Van de Walle.^[^ ^56^, ^57^ ^]^

## Conflict of Interest

The authors declare no conflict of interest.

## Supporting information

Supporting Information

## Data Availability

The data that support the findings of this study are available from the corresponding author upon reasonable request.
