# Supplementary material for: Photoluminescence Intensity Enhancement in Tin Halide Perovskites
Source: Adv Sci (Weinh). 2022 Sep 15;9(32):2202795. doi: 10.1002/advs.202202795 (PMC9661860; doi:10.1002/advs.202202795)
Supplement: Supplementary file 1 — Supporting Information [file ADVS-9-2202795-s001.pdf]

ADVANCED SCIENCE
Open Access

Supporting Information

for *Adv. Sci.*, DOI 10.1002/advs.202202795

Photoluminescence Intensity Enhancement in Tin Halide Perovskites

*Isabella Poli, Francesco Ambrosio, Antonella Treglia, Felix J. Berger, Mirko Prato, Munirah D. Albaqami, Filippo De Angelis and Annamaria Petrozza**

Supporting Information

Photoluminescence Intensity Enhancement in Tin Halide Perovskites

Isabella Poli[1] , *Francesco Ambrosio*[1,3,6] , *Antonella Treglia*[1,2], *Felix J. Berger*[1], *Mirko Prato*[7], *Munirah D. Albaqami*[5] , *Filippo De Angelis*[3,4], *Annamaria Petrozza*[1,5*]

1. Center for Nano Science and Technology @PoliMi, Istituto Italiano di Tecnologia, via G. Pascoli 70/3, 20133, Milano, Italy.

2. Physics Department, Politecnico di Milano, Piazza L. da Vinci, 32, 20133 Milano, Italy.

3. Computational Laboratory for Hybrid/Organic Photovoltaics (CLHYO), Istituto CNR di Scienze e Tecnologie Chimiche "Giulio Natta" (CNR- SCITEC), Perugia, Italy.

4. Department of Chemistry, Biology and Biotechnology, University of Perugia, Perugia, Italy.

5. Chemistry Department, College of Science, King Saud University, Riyadh 11451, Saudi Arabia

6. Department of Chemistry and Biology "A. Zambelli", University of Salerno, Via Giovanni Paolo II 132, 84084 Fisciano, Salerno, Italy.

7. Materials Characterization Facility, Istituto Italiano di Tecnologia, Via Morego 30, 1613 Genova

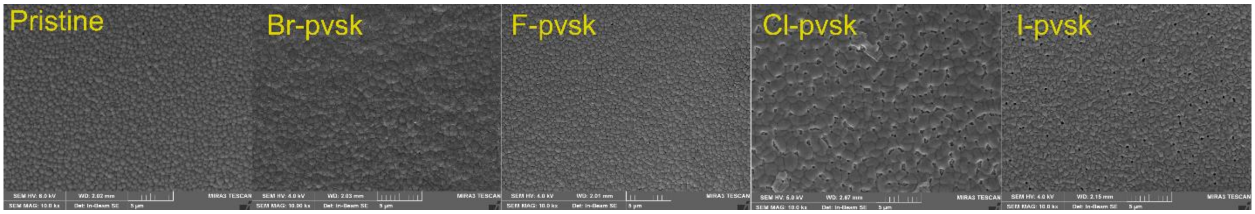

Figure S 1. Top-view SEM images of pristine and doped FACsSnI films. Scale bar = 5 µm.

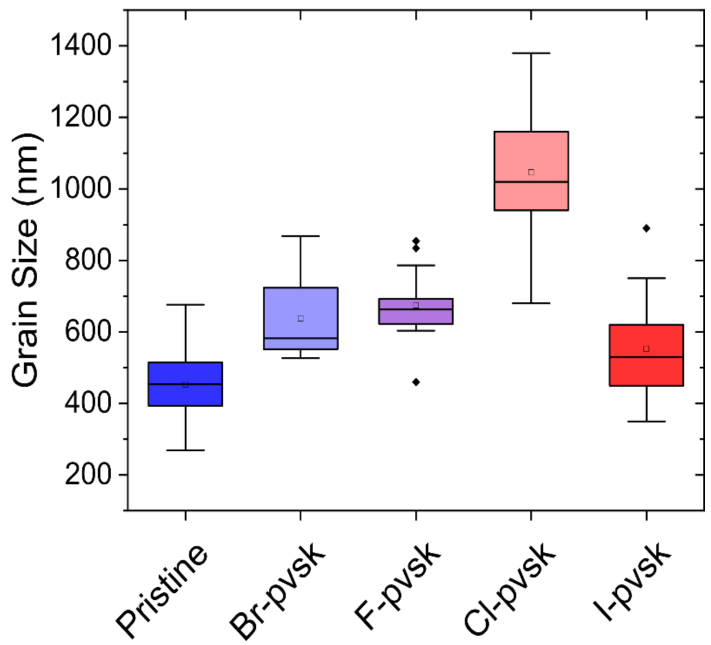

Figure S 2. Grain size of FACsSnI films without and with extra SnX_2 measured via top view SEM images

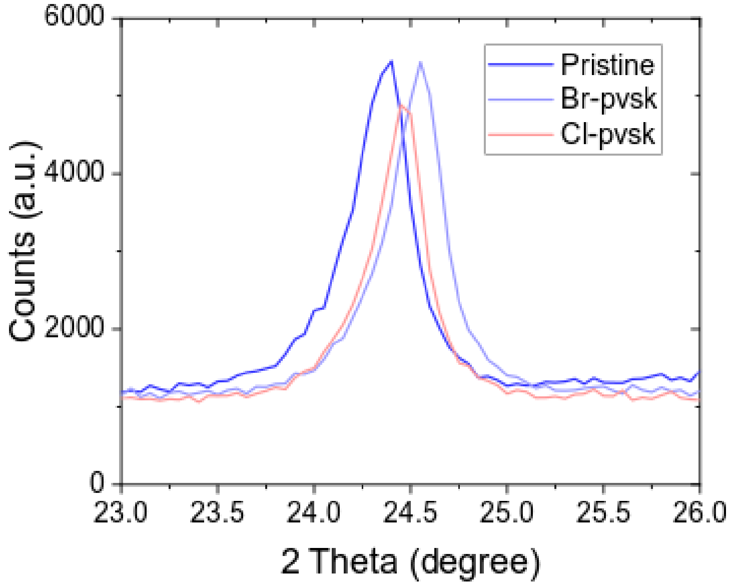

Figure S 3. XRD (111) reflection peak of pristine and Br- and Cl-pvsk FACsSnI thin films.

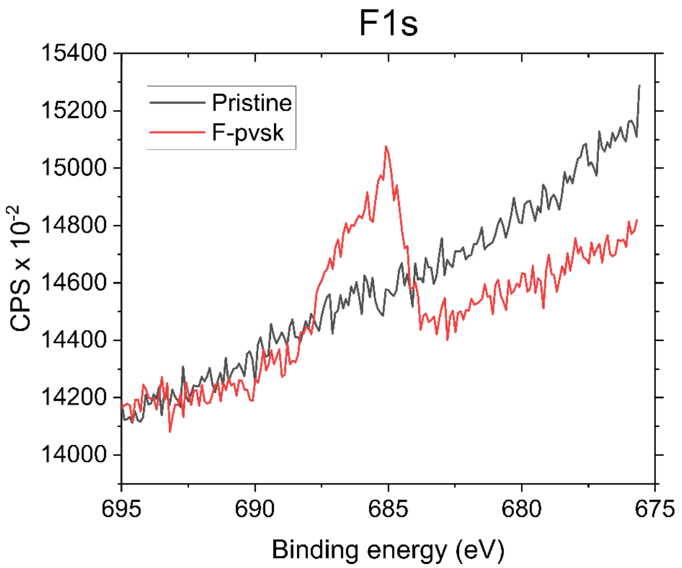

Figure S 4. F1s spectra of pristine (grey) and F-pvsk (red) FACsSnI thin films.

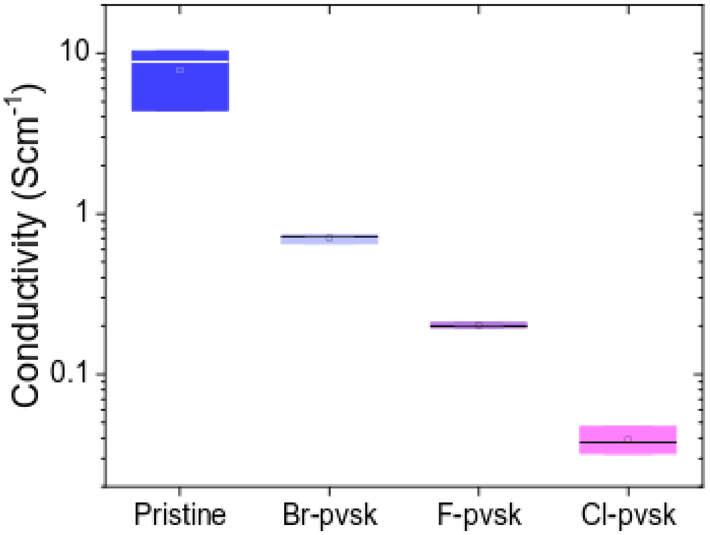

Figure S 5. Conductivity of FACsSnI thin films without and with excess SnX_2 measured in the dark in the glovebox on films that have never been exposed to oxygen air.

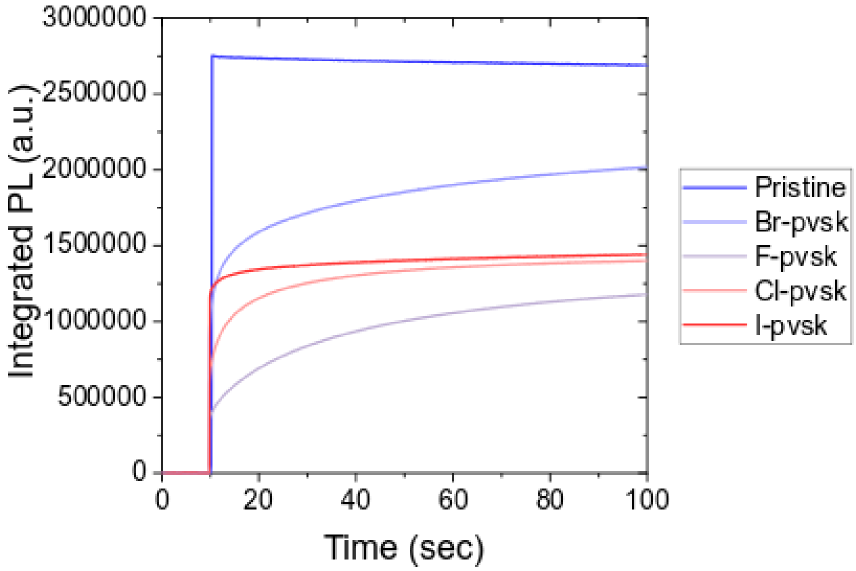

Figure S 6. Integrated photoluminescence over time of FACsSnI films without and with extra SnX_2. Excitation source is 450 nm at 100 mWcm^{-2}

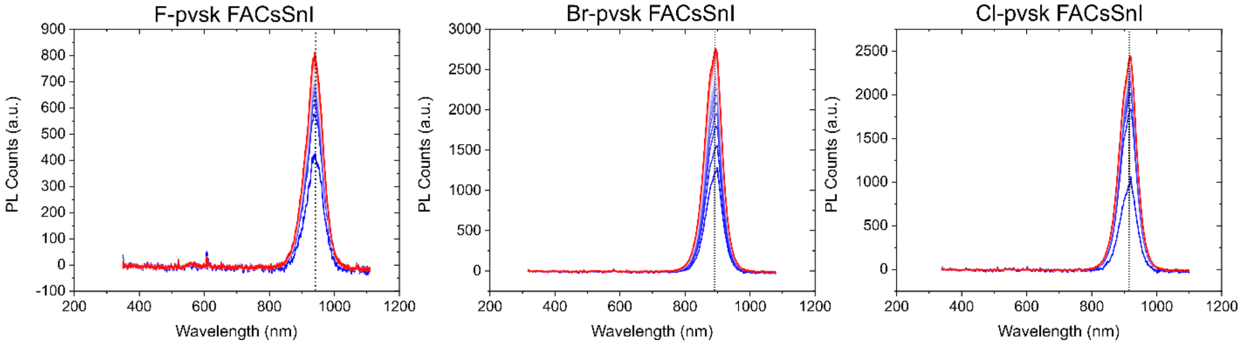

Figure S 7. PL spectra of F-pvsk, Br-pvsk and Cl-pvsk FACsSnI thin films under continuous excitation for 600 second (time goes from blue-0 sec to red-600 sec).

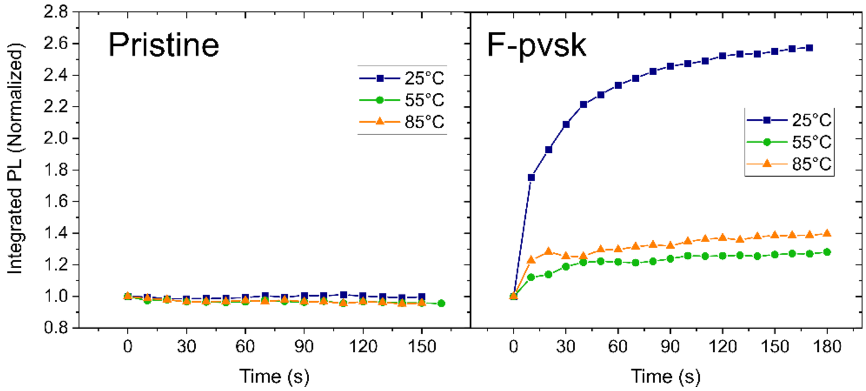

Figure S 8. Integrated PL evolution of pristine and F-pvsk FACsSnI films under continuous excitation (cw 520 nm) for 3 minutes while the substrates are kept at 25, 55 and 85°C.

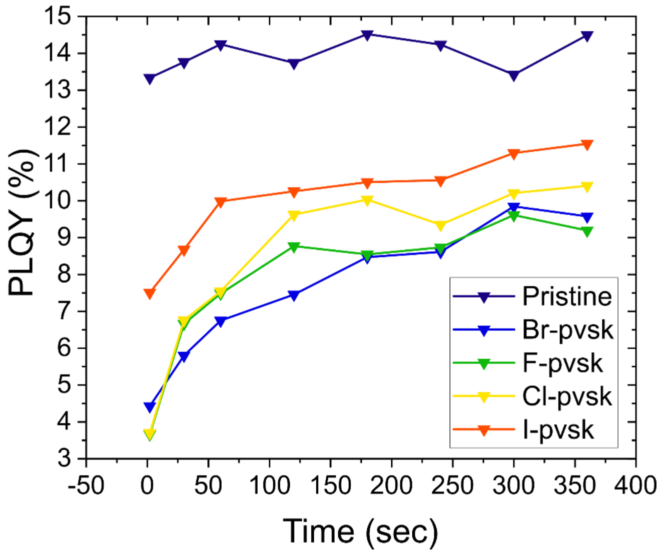

Figure S 9. Absolute PLQY values of FACsSnI thin films without and with extra SnX_2 measured under 100 $mWcm^{-2}$ with an excitation of 375 nm in an integrating sphere. The samples measured here and in Figure 2a comes from different batches.

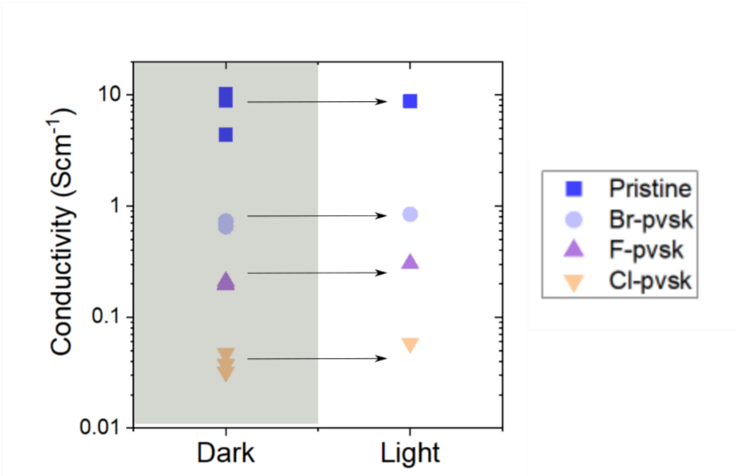

Figure S 10. Conductivity of FACsSnI thin films without and with extra SnX_2 measured in the same spot in the dark (left) and after 3 minute of illumination (right).

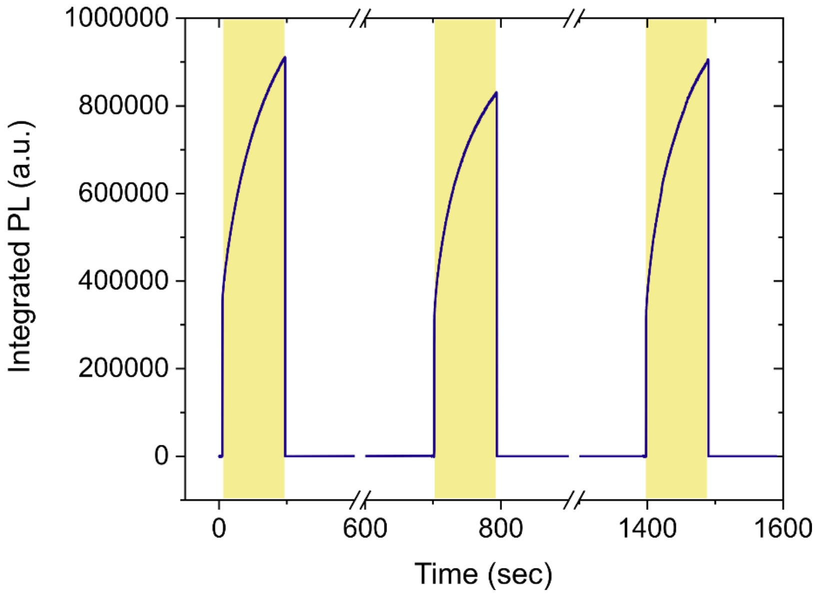

Figure S 11. Integrated photoluminescence of a Br-pvsk FACsSnI thin film measured under continuous illumination for three consecutive times with an excitation of 450 nm. Between each measurement, the sample was kept in the dark for 10 minutes.

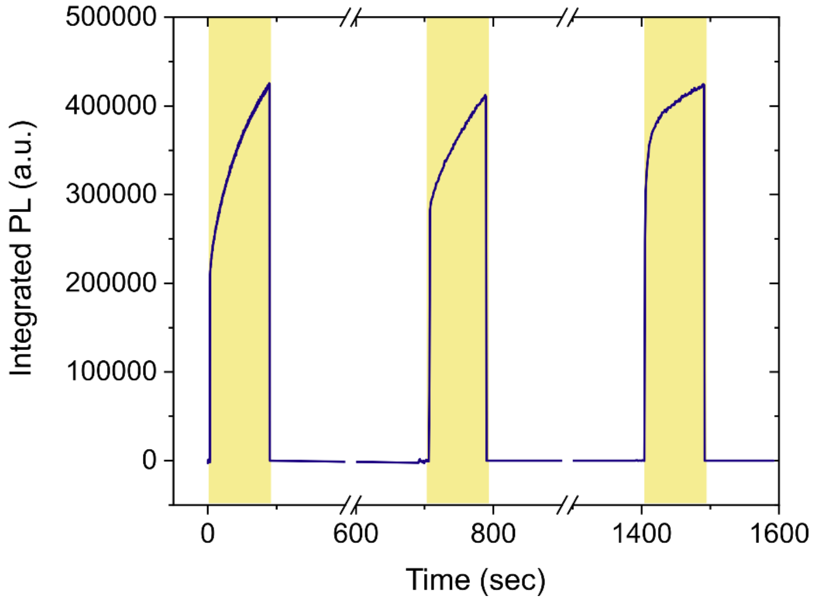

Figure S 12. Integrated photoluminescence of a Cl-pvsk FACsSnI thin film measured under continuous illumination for three consecutive times with an excitation of 450 nm. Between each measurement, the sample was kept in the dark for 10 minutes.

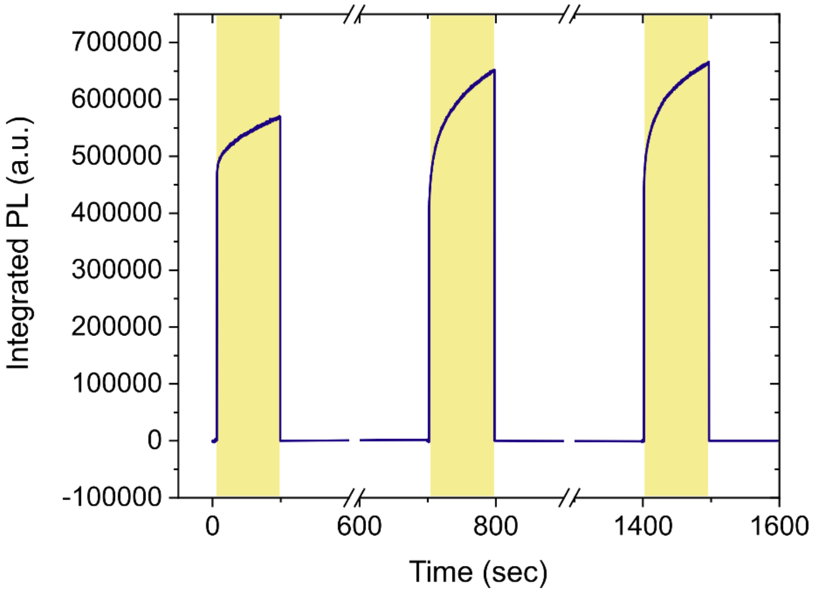

Figure S 13. Integrated photoluminescence of a I-pvsk FACsSnI thin film measured under continuous illumination for three consecutive times with an excitation of 450 nm. Between each measurement, the sample was kept in the dark for 10 minutes.

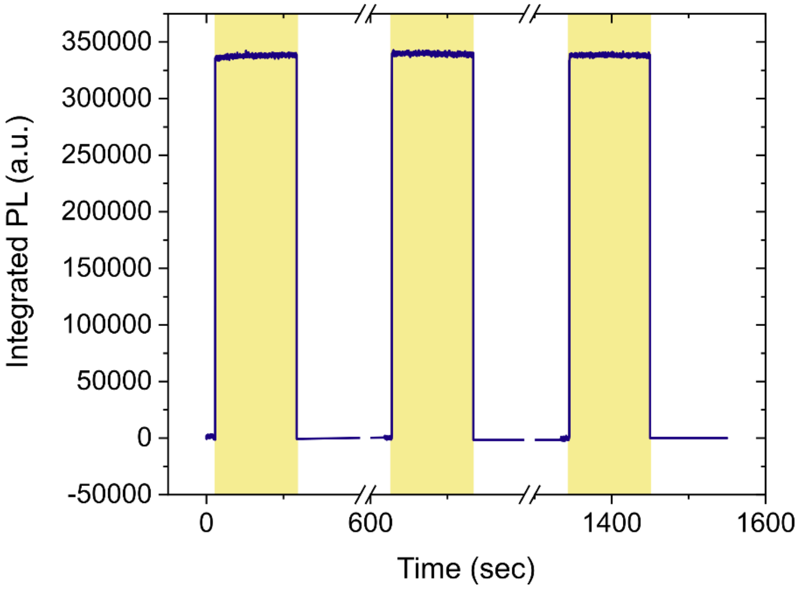

Figure S 14. Integrated photoluminescence of a pristine FACsSnI thin film measured under continuous illumination for three consecutive times with an excitation of 450 nm. Between each measurement, the sample was kept in the dark for 10 minutes.

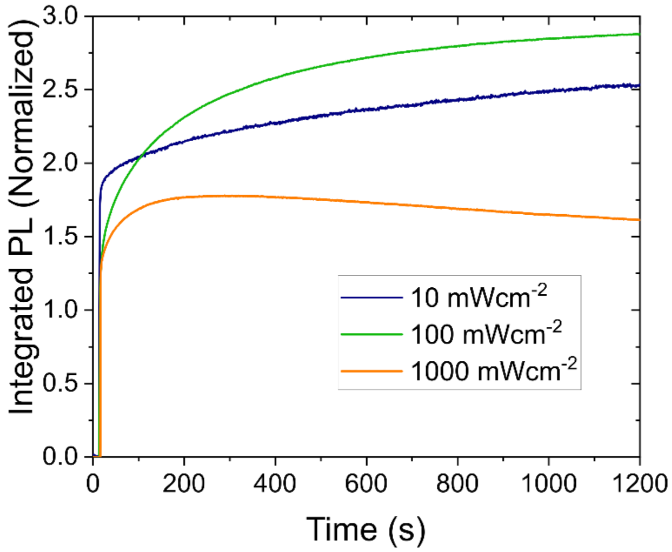

Figure S 15. Integrated photoluminescence intensity of a Br-pvsk FACsSnI thin film excited with 450 nm light source at 10, 100 and 1000 mWcm^{-2} power intensities. Data have been normalized with respect to the initial integrated PL intensity value measured as soon as the light source is turned on.

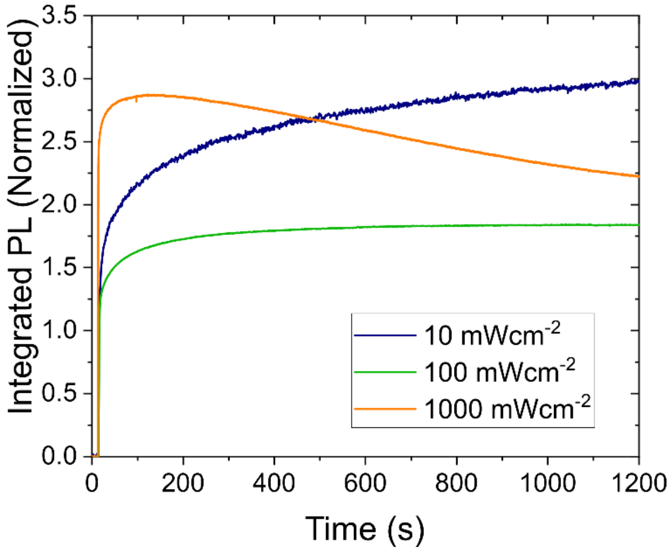

Figure S 16. Integrated photoluminescence intensity of a Cl-pvsk FACsSnI thin film excited with 450 nm light source at 10, 100 and 1000 mWcm^{-2} power intensities. Data have been normalized with respect to the initial integrated PL intensity value measured as soon as the light source is turned on.

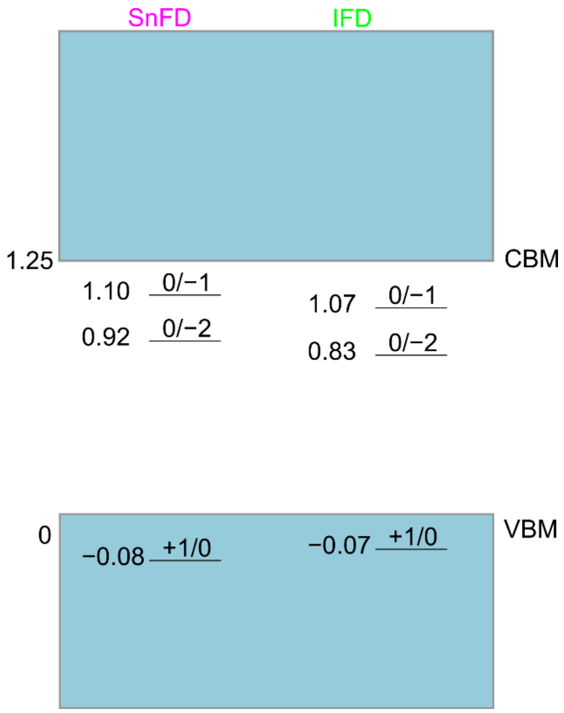

Figure S 17 Thermodynamic charge transition levels for the Sn and I Frenkel defects (SnFD and IFD) in tetragonal $MASnI_3$. Energies are given in eV and are referred to that of the valence band edge of the pristine material.
